# Evaluation of phenotypic and genotypic methods for detecting KPC variants

**DOI:** 10.1128/aac.00082-25

**Published:** 2025-04-03

**Authors:** Yasmine Benhadid-Brahmi, Claire Amaris Hobson, Lydia Abdelmoumene, Ella Jaouen, Mélanie Magnan, Maud Gits-Muselli, Mathilde Lescat, Olivier Tenaillon, Stéphane Bonacorsi, André Birgy

**Affiliations:** 1 IAME, UMR 1137, INSERM, Université Paris Cité 555089, Paris, Île-de-France, France; 2 Service de Microbiologie, Hôpital Robert-Debré, AP-HP, Paris, France; 3 Service de Maladies Infectieuses et Tropicales, Hôpital Bichat, AP-HP, Paris, France; 4 Bacteriology Unit, CNR le Charbon, Microbiology and Infectious Diseases Department, Institut de Recherche Biomédicale des Armées 195617 , Brétigny-sur-Orge, Île-de-France, France; University of Fribourg, Fribourg, Switzerland

**Keywords:** KPC beta-lactamase, clinical KPC variants, detection tests, LFIA tests, hydrolysis-based tests

## Abstract

*Klebsiella pneumoniae* carbapenemases (KPCs) have spread and diversified extensively. To date, 242 clinical variants have been identified and harbor different hydrolytic capacities, thereby interfering with rapid diagnostic tests. The accurate detection of KPC variants is crucial to guide treatment and control measures in healthcare settings. We constructed KPC variants to assess the mutational impact on detection capacities of resistance-based tests. KPC variants (*n* = 45) were characterized phenotypically and used to measure the detection sensitivity of KPC detection methods (two lateral flow immunoassays [LFIAs], three hydrolysis tests, three selective culture media, and two PCR-based tests). We identified four antibiotic susceptibility patterns: “KPC-like” (23/45; 51%), “extended-spectrum beta-lactamase-like” (6/45; 13%), “ceftazidimase” (9/45; 20%), and outlier variants with “mixed-profiles” (5/45; 11%). These phenotypes had different impacts on the detection capabilities of hydrolysis tests (0%–100%), LFIA (44%–100%), and selective culture media (0%–100%), highlighting a risk of misdiagnosis for some KPC variants. All variants were detected with PCR-based tests. To detect the maximum of KPC variants, fecal carriage screening requires a combination of selective media targeting resistance to carbapenems, third-generation cephalosporins, and ceftazidime-avibactam. From antibiotic susceptibility testing, resistance to ceftazidime ± avibactam and specific phenotypic profiles should be used as warnings to track the presence of KPC variants. We recommend LFIA as a first-line test, owing to its high sensitivity in detecting KPC variants. Nevertheless, using a combination of tests may remain wise in some situations. The spread of KPC variants remains a significant concern, particularly as reversion to ancestral phenotype could restore carbapenem resistance and lead to therapeutic failure

## INTRODUCTION

*Klebsiella pneumoniae* carbapenemase (KPC) is a prevalent carbapenem resistance mechanism among Enterobacterales in various countries, including those of South America, the USA, India, Mediterranean countries, and Europe (1–3). KPC-2 and KPC-3 are the most frequent such enzymes (2). KPCs confer broad resistance to penicillins, cephalosporins, aztreonam, and carbapenems, but also to standard beta-lactamase inhibitors (clavulanic acid, tazobactam, and sulbactam). New beta-lactamase inhibitors (avibactam, vaborbactam, and relebactam) are effective against KPC beta-lactamase (4, 5) and have been shown to be effective against KPC-producing Enterobacterales, resulting in lower mortality *in vivo* (4, 6).

KPC beta-lactamases have diversified extremely rapidly under antibiotic selection pressure, with this diversification potentially enhanced by the increasing use of ceftazidime-avibactam. As of January 2025, 242 different variants had been identified in clinical contexts, with a significant proportion showing resistance to ceftazidime-avibactam (at least 40%–50%), highlighting the remarkable adaptability and evolutionary capabilities of this enzyme (2).

Insertions, deletions, and point mutations may occur throughout the KPC gene, but most are located in the three loops surrounding the active site (the omega-loop_164-179_, Loop_238–243_, and Loop_267–275_) (7). These genetic modifications can lead to changes in the ability of the protein to hydrolyze beta-lactam antibiotics, resulting in heterogeneous levels of resistance to carbapenems, third-generation cephalosporins, penicillins, and beta-lactamase inhibitors. Various phenotypes may therefore be observed, including the three main phenotypes: the “KPC-like” phenotype with polyvalent activity against beta-lactams, the “extended-spectrum beta-lactamase-like” (“ESBL-like”) phenotype, and the “ceftazidimase” phenotype with highly specialized activity against ceftazidime (2, 3, 7). These modifications render the phenotypic detection of these variants more complicated.

However, *in vivo* reversion to the initial phenotype (carbapenem resistance) is possible, so accurate detection is essential to guide therapeutic choices and control measures in healthcare settings (8–10). We investigated the correlation between resistance phenotype and the ability of screening or diagnostic tests (selective culture media, lateral flow immunoassays (LFIAs), beta-lactam hydrolysis tests, and molecular detection) to detect the variant, on a collection of 45 KPC variants.

## RESULTS

### Strains, antibiotic susceptibility testing, and classification of KPC variants based on their profiles

The selected variants encompass most of the residues frequently mutated in clinical KPC variants. The mutational hotspots of KPC beta-lactamases are mostly clustered around the three loops near the active site, and the most frequently mutated residues include D179, A172, L169, T243, and R164, mutated in 39 (16%), 11 (4.5%), 11 (4.5%), 9 (3.7%), and 8 (3.2%) of clinical variants, respectively. Due to their high mutation frequency, these residues are more likely to be mutated in clinical variants and therefore to appear in clinical settings, hence the importance of evaluating resistance-based tests. In addition, insertions and deletions involving the omega-loop_164-179_, Loop_238–243_, or Loop_267–275_ are frequent. They are observed in 34 (14%), 12 (5%), and 94 (38.5%) of variants, respectively. If all three loops are considered together, insertions or deletions are found in almost 50% of clinical KPC variants.

These mutations have different effects on the hydrolytic capacities of the protein. Of the 45 variants tested, 36 (80%) were resistant to amoxicillin, 37 (82%) to cefotaxime, 44 (98%) to ceftazidime, 18 (40%) to ceftazidime-avibactam, and 20 (44%) to cefepime, whereas 29 (64%) retained the ability to hydrolyze carbapenems (imipenem, meropenem, or ertapenem).

**Fig 1:**
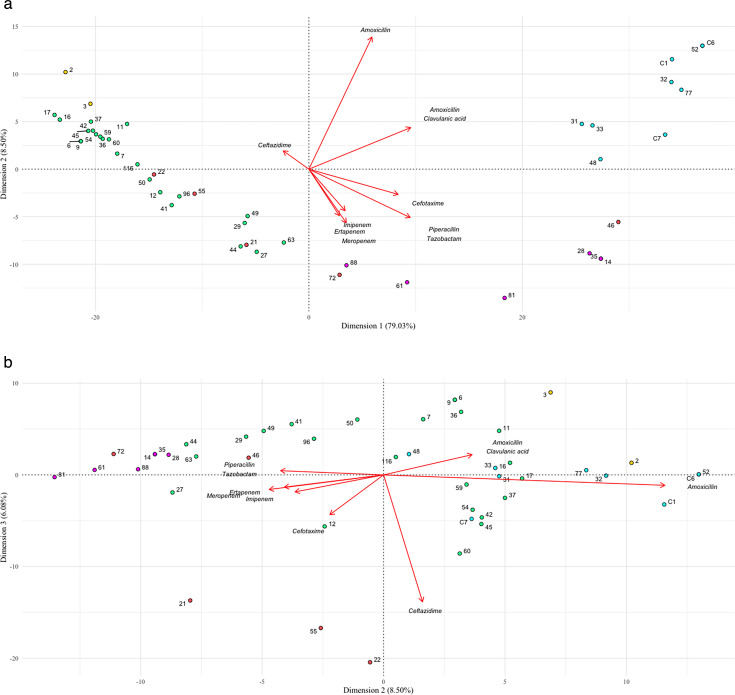
PCA on the inhibition zone diameters of representative beta-lactams for the 45 strains studied. Each point is labeled with the strain indicated in Table 1. The clusters “ancestral KPC,” “KPC-like,” “ESBL-like,” and “ceftazidimase” are shown in yellow, green, pink, and blue, respectively, and the “mixed” cluster (21, 22, 46, 55, and 72) is shown in red. Arrows indicate higher susceptibility to antibiotics. The first two principal components explained 79.03% (dimension 1—Fig. 1a) and 8.5% (dimension 2—Fig. 1b) of the total variance.

**Fig 2:**
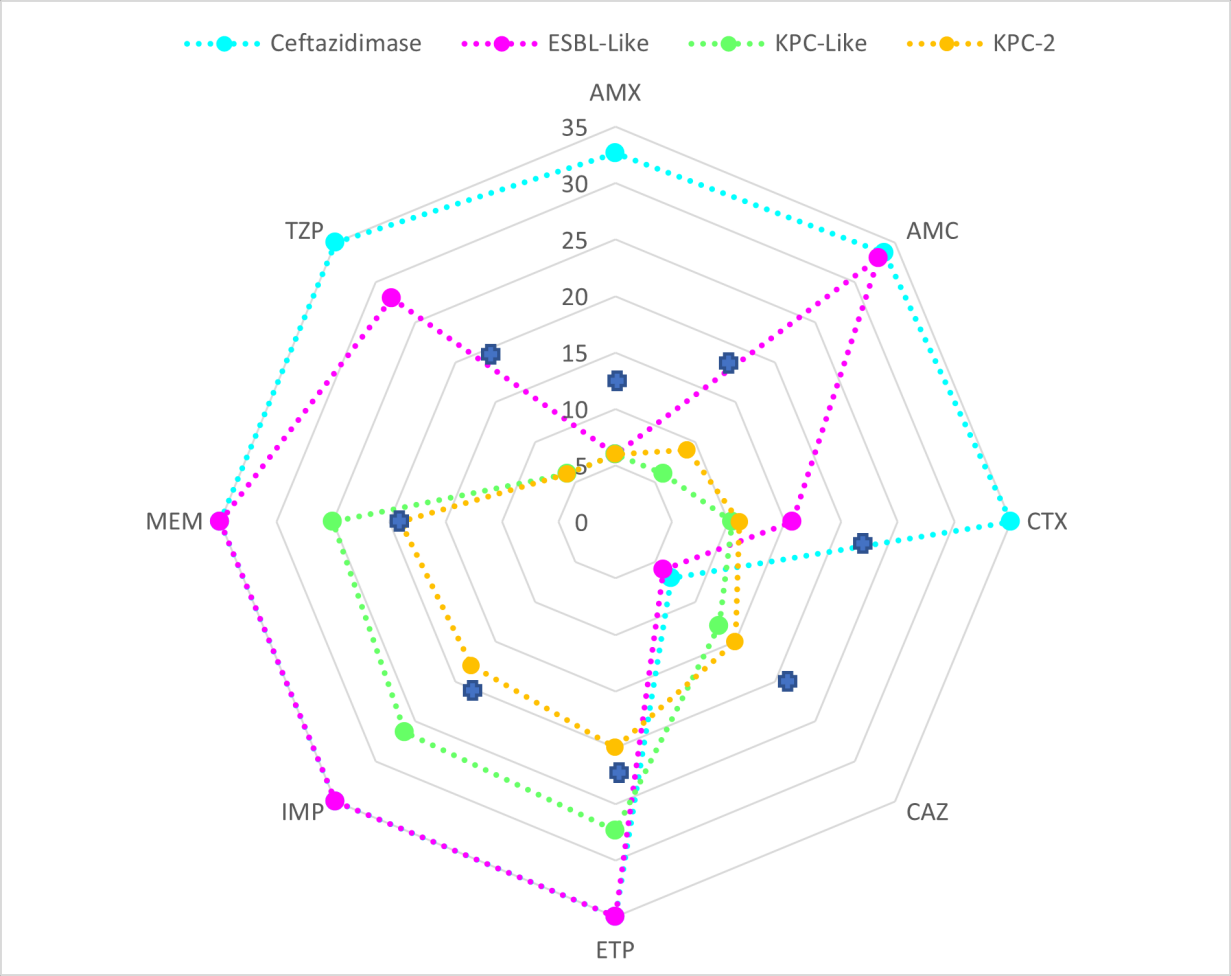
Kiviat diagram showing the distribution of mean inhibition zone diameters for the principal antibiotic molecules tested, by major phenotypic profile: “ancestral KPC,” “KPC-like,” “ceftazidimase,” and “ESBL-like.” AMX, amoxicillin; TZP, piperacillin-tazobactam; CTX, cefotaxime; CAZ, ceftazidime; ETP, ertapenem; IMP, imipenem; MEM, meropenem. Diameters: millimeters; blue crosses: EUCAST breakpoints (11).

We further explored the functional impact of these structural modifications by performing a principal component analysis (PCA) on the inhibition zone diameters for representative beta-lactams for 45 variants. We identified distinct clusters corresponding to “ancestral KPC,” “KPC-like,” “ESBL-like,” “ceftazidimase,” and five outlier strains (KPC-21, 22, 46, 55, and 72) grouped together as “mixed.” Projections of the variables onto dimensions 1 and 2 and dimensions 2 and 3 are shown in Fig. 1. The first two principal components explained 79.03 and 8.50% of the total variance, respectively, highlighting positive correlations between susceptibilities to carbapenems, piperacillin-tazobactam, and cefotaxime, and a separate correlation between susceptibilities to amoxicillin and amoxicillin-clavulanate. A negative correlation was observed between resistances to ceftazidime and carbapenems, piperacillin-tazobactam, and cefotaxime. The representation of mean inhibition zone diameters on Fig. 2 confirms the identification of the main phenotypes.

**TABLE 1:** Phenotypic description of 45 KPC beta-lactamase variants, including MIC determination for AMX (amoxicillin), PTZ (piperacillin-tazobactam), CTX (cefotaxime), CTX/CLAV (cefotaxime-clavulanic acid), CAZ (ceftazidime), CAZ/CLAV (ceftazidime-clavulanic acid), CZA (ceftazidime-avibactam), FEP (cefepime), IMI (imipenem), MERO (meropenem), and ETP (ertapenem)^*a*^^*b*^

		Position of mutation in enzyme structure				MIC (mg/L)										
**Isolate/variant (Number of variants with this residue/loop mutated (total= 243))**	Mutation*	**Omega-loop** **164-179**	**Loop 238-243**	**Loop** **267-275**	**Other site**	**AMX**	**PTZ**	**CTX**	**CTX/CLAV 4:1**	**CAZ**	**CAZ/CLAV 4:1**	**CZA 4:1**	**FEP**	**IMI**	**MERO**	**ETP**
Breakpoints MIC (R > x)						8	8	2	2	4	4	8	4	4	8	0.5
*E. coli* TOP10 : Wild type						-	-	-	-	-	-	0.06	-	<0.5	-	0.012
*E. coli* ATCC25922 : Wild type						-	-	-	-	-	-	0.25	-	<0.5	-	0.008
KPC-2 : Ancestral KPC						>16	>64	32	2	16	16	0.5	8	2	4	>1
KPC-3 : Ancestral KPC	*					>16	>64	16	4	64	32	1	8	2	4	1
KPC-44 : KPC-Like (94)	ins261_AVYTRAPNKDDKHSE *			+		>16	>64	16	2	>128	8	16	8	2	0.5	0.5
KPC-50 : KPC-Like (94)	ins275_EAV *			+		>16	>64	8	1	>128	32	>32	8	2	0.5	0.25
KPC-41 : KPC-Like (94)	ins267_PNK *			+		>16	>64	32	1	>128	32	32	16	2	<0.12	0.12
KPC-29 : KPC-Like (94)	ins269_KDD *			+		>16	64	8	0.5	>128	8	32	16	2	0.25	0.12
KPC-59 : KPC-Like (1)	G89D				+	>16	>64	32	4	32	16	0.5	16	4	2	>1
KPC-45 :KPC-Like (2)	T93K				+	>16	>64	32	4	32	8	<0.5	8	4	2	>1
KPC-6 : KPC-Like (7)	V240G		+			>16	>64	>64	4	128	8	2	8	4	2	0.5
KPC-11 : KPC-Like (6)	P104L				+	>16	>64	4	0.5	16	<0.12	<0.5	2	<0.5	0.25	0.25
KPC-27 : KPC-Like (3)	W105R *				+	>16	32	32	16	64	64	1	<0.5	2	<0.12	0.12
KPC-60 : KPC-Like (2)	A1118T				+	>16	>64	16	4	16	4	<0.5	8	4	2	1
KPC-36 : KPC-Like (2)	D163E *				+	>16	>64	32	4	128	32	1	>16	4	2	1
KPC-49 : KPC-Like (8)	R164S *	+				>16	32	32	0.5	>128	8	8	16	2	0.25	0.5
KPC-12 : KPC-Like (11)	L169M	+				>16	64	8	0.5	32	4	<0.5	4	1	0.25	0.25
KPC-17 : KPC-Like (4)	F207L				+	>16	>64	32	16	16	16	<0.5	16	2	2	1
KPC-09 : KPC-Like (7)	V240A *		+			>16	>64	>64	16	>128	128	2	>16	2	2	0.5
KPC-63 : KPC-Like (5)	Y241S *		+			>16	64	8	<0.12	128	32	16	4	2	2	1
KPC-42 : KPC-Like (1)	T254A				+	>16	>64	32	2	16	8	<0.5	8	4	4	>1
KPC-7 : KPC-Like (1)	M49I *				+	>16	>64	32	4	128	2	1	16	4	4	>1
KPC-54 : KPC-Like (1)	A62S				+	>16	>64	32	2	8	16	<0.5	4	4	4	>1
KPC-96 : KPC-Like (5)	Y241N		+			>16	>64	16	0.5	64	8	16	4	2	1	1
KPC-116 : KPC-Like (5)	S171F	+				>16	>64	16	1	32	4	1	4	2	0.5	0.25
KPC-16 : KPC-Like (1 and 4)	P202S F207L				+	>16	>64	32	4	32	8	<0.5	8	4	4	>1
KPC-37 : KPC-Like (2 and 4)	W165R F207L	+			+	>16	>64	32	8	32	<0.12	<0.5	4	2	2	1
KPC-28 : KPC-Like (12)	del242-243_GT *		+			>16	<4	8	<0.12	>32	4	32	4	<0.5	<0.12	0.25
KPC 14 : ESBL-Like (12)	del242-243_GT		+			>16	<4	16	<0.12	>128	4	32	8	<0.5	<0.12	<0.06
KPC-81 : ESBL-Like (34)	del1731I	+				>16	4	32	<0.12	>128	2	16	>16	<0.5	<0.12	0.12
**Isolate/variant (Number of variants with this residue/loop mutated (total= 243))**	Mutation*	**Omega-loop** **164**-**179**	**Loop 238**-**243**	**Loop** **267**-**275**	**Other site**	**AMX**	**PTZ**	**CTX**	**CTX/CLAV** **4:1**	**CAZ**	**CAZ/CLAV 4:1**	**CZA** **4:1**	**FEP**	**IMI**	**MERO**	**ETP**
KPC-61 : ESBL-Like (5)	S171P *	+				>16	<4	2	<0.12	>128	<0.12	8	2	<0.5	<0.12	0.12
KPC-35 : ESBL-Like (11)	L169P	+				>16	<4	4	<0.12	>128	2	4	2	<0.5	<0.12	<0.12
KPC-88 : ESBL-Like (2)	D176Y	+				>16	16	4	<0.12	64	2	8	1	<0.5	<0.12	0.12
C1 non clinical : Ceftazidimase (34)	del165-175_WELELNSAIPG *	+				<8	<4	0.5	<0.12	64	16	16	<1	<0.5	<0.12	<0.06
C6 non clinical : Ceftazidimase (34)	del168-171_ELNS *	+				<8	<4	2	<0.12	128	4	>32	1	<0.5	<0.12	<0.06
C7 non clinical : Ceftazidimase (34)	del168-169_EL	+				<8	<4	1	<0.12	64	0.5	1	1	<0.5	<0.12	<0.06
KPC-52: Ceftazidimase(39 and 94)	D179Y ins262_V	+		+		<8	<4	1	<0.12	>128	32	>32	1	<0.5	<0.12	<0.06
KPC-77 : Ceftazidimase (8)	R164P	+				<8	<4	1	<0.12	128	2	8	<1	<0.5	<0.12	<0.06
KPC-31 : Ceftazidimase (39)	D179Y *	+				16	<4	4	<0.12	>128	4	>32	4	<0.5	<0.12	<0.06
KPC-33 : Ceftazidimase (39)	D179Y	+				16	<4	4	<0.12	>128	2	16	2	<0.5	<0.12	<0.06
KPC-32 : Ceftazidimase(39 and 9)	D179Y T243M *	+	+			<8	8	4	<0.12	128	4	32	4	<0.5	<0.12	0.12
KPC-48 : Ceftazidimase(11 and 11)	L169P A172T	+				16	<4	4	<0.12	>128	4	16	2	<0.5	<0.12	<0.06
KPC-21 : Mixed (3)	W105R				+	>16	16	32	16	16	16	<0.5	<0.5	2	<0.12	<0.06
KPC-46 : Mixed (11)	L169P *	+				>16	<4	16	<0.12	>128	4	16	8	<0.5	<0.12	<0.06
KPC-72 : Mixed (12)	A172D	+				>16	32/	8	1	>32	>128	16	2	1	<0.12	0.12
KPC-55 : Mixed (2)	Y264N				+	>16	>64	4	0.5	8	1	<0.5	4	4	4	1
KPC-22 : Mixed (3 and 4)	W105G F207L				+	>16	>64	8	4	4	4	<0.5	2	2	2	0.25

^*a*^ MIC, minimum inhibitory concentration.

^*b*^ + : presence , – : absence, * Presence of mutation H274Y.

“Ancestral KPCs” were resistant to most beta-lactams, including carbapenems and ceftazidime but susceptible to ceftazidime-avibactam.

The “KPC-like” profile, observed in 23 of 45 variants (51%), resembles ancestral enzymes with resistance to ceftazidime, and 6 of 23 variants (26%) were resistant to ceftazidime-avibactam, but with a higher susceptibility to carbapenems.

The “ESBL-like” profile, identified in 6 of 45 strains (13%), displayed resistance to amoxicillin and cefotaxime, but strong synergy with beta-lactamase inhibitors. These variants were also resistant to ceftazidime, with 3 of 6 resistant to ceftazidime-avibactam, and they were susceptible to carbapenems.

The “ceftazidimase” profile, present in 9 of 45 variants (20%), corresponded to highly specialized resistance to ceftazidime, with susceptibility to other beta-lactams, including amoxicillin and cefotaxime (Table 1 and Fig. 2). The specialized resistance of these variants to ceftazidime may result in weaker inhibition by avibactam, leading to ceftazidime-avibactam resistance, as observed in 7 of 9 variants (12). This profile included variants with either deletions or point mutations in the omega-loop.

Finally, the outlier strains grouped together as the “mixed” cluster displayed various phenotypes: KPC-46 and KPC-72 were resistant to amoxicillin, ceftazidime, and ceftazidime-avibactam but susceptible to cefotaxime, carbapenems, and piperacillin-tazobactam. Projections onto dimensions 2 and 3 (Fig. 1) identified outliers KPC-21, KPC-22, and KPC-55, with phenotypes similar to the “KPC-like” profile but greater susceptibility to ceftazidime and cefotaxime, together with susceptibility to ceftazidime-avibactam. Details about the mutations, minimum inhibitory concentrations (MICs), phenotypic profiles, and inhibition zone diameters are provided in Table 1, Fig. 2, and Table S2.

### Rapid diagnostic tests

#### LFIA

For the 45 KPC variants tested, 40 (88%) and 39 (87%) were correctly detected by Resist-5 O.K.N.V.I and NG-Test Carba 5, respectively. Five strains were not detected by either of the two tests. These three strains all had a “ceftazidimase” profile with point mutations (KPC-31, KPC-32, KPC-33, and KPC-77) or a deletion (C1) in the omega-loop. KPC-37 was correctly detected by the Resist-5 O.K.N.V.I test but not by the NG-Test Carba 5. This strain had a “KPC-like” profile, with a double mutation in the omega-loop (Table 2 and Table S3).

**TABLE 2:** Sensitivity of detection tests based on phenotypic profiles and their applicability

Phenotypic profile	Number of phenotypes (Sensitivity %)									
	Resistance based-test								Molecular tests	
	Resist-5 O.K.N.V.I	NG-Test Carba 5	Beta Carba test	RAPIDEC Carba NP	Beta Lacta Test	ChromID Carba smart	ChromID ESBL	Chromatic Super CAZ/AVI	Xpert Carba-R assay	BioFire Filmarray Phenotypic
Ancestral KPC (n=2)	2 (100%)	2 (100%)	2 (100%)	2 (100%)	2 (100%)	2 (100%)	2 (100%)	0	2 (100%)	2 (100%)
KPC-Like (*n* = 23)	23 (100%)	22 (96%)	20 (87%)	16 (70%)	23 (100%)	23 (100%)	23 (100%)	7 (30%)	23 (100%)	23 (100%)
ESBL-Like (*n* = 6)	6 (100%)	6 (100%)	0	0	6 (100%)	0	6 (100%)	6 (100%)	6 (100%)	6 (100%)
Ceftazidimase (*n* = 9)	4 (44%)	4 (44%)	0	0	0	0	0	8 (89%)	9 (100%)	9 (100%)
Mixed (*n* = 5)	5 (100%)	5 (100%)	3 (60%)	3(60%)	5 (100%)	4 (80%)	4 (80%)	2 (40%)	5 (100%)	5 (100%)
All profiles (*n* = 45)	40 (88%)	39 (87%)	25 (56%)	21 (47%)	36 (80%)	29 (64%)	35 (78%)	23 (51%)	45 (100%)	45 (100%)
Usable on strains with the following phenotypes	All but possible false negative results with "ceftazidimase" variants		KPC-Like and Mixed		All but ceftazidimase	KPC-Like and Mixed	All but ceftazidimase	Only on CAZ/AVI resistant variants	All	

#### Hydrolysis-based tests

We were able to detect 25 (56%) of the 45 KPC variants tested with the Beta Carba test, and 21 (47%) with the Rapidec Carba NP test. These variants had either a “KPC-like” or a “mixed” profile. Twenty of the 24 variants not detected by the Rapidec Carba NP test were also not detected by the Beta Carba test. Fifteen of the strains missed by both tests had ESBL-like or “ceftazidimase” profiles, whereas the other five had a “KPC-like” or “mixed” profile. These last five strains were correctly identified both by culture on ChromID Carba Smart and in both LFIA tests.

The Beta Lacta test detected 36 (80%) of the 45 strains tested, all of which had a “KPC-like,” “mixed,” or “ESBL-like” profile. The nine strains not detected by the Beta Lacta test had a “ceftazidimase” profile. These strains also failed to grow on both ChromID Carba Smart and ChromID ESBL selective media (Table 2 and Table S3).

### Sensitivity of detection on selective chromogenic agar media

Of the 45 KPC variants, 29 (64%) grew on both ChromID Carba Smart and ChromID ESBL media. Six of the 16 variants that did not grow on ChromID Carba Smart selective medium had an “ESBL-like” profile and grew on ChromID ESBL, whereas 10 had a “ceftazidimase” or “mixed” profile and did not grow on ChromID ESBL medium (Table 2 and Table S3). In total, 23 of the 45 KPC variants (all having a ceftazidime/avibactam MIC ≥4 mg/L) grew on Chromatic Super CAZ/AVI: 7 of 23 of “KPC-like,” 6 of 6 “ESBL-like,” 8 of 9 “ceftazidimase,” and 2 of 5 “mixed” profiles (Table 2).

The same results were observed with inocula of 10² and 10⁵ CFU.

#### PCR-based test

Both molecular detection methods were able to detect all the variants with a sensitivity of 100% (Table 2 and Table S3).

## DISCUSSION

The rapid diversification of KPC beta-lactamases has led to a wide range of hydrolytic and resistance profiles. Some mutations lead to a resistance profile similar to that of the ancestral enzyme with a reduction of carbapenemase activity, whereas others result in more specialized profiles with frequent resistance to ceftazidime ± avibactam. In our collection, resistance to ceftazidime and ceftazidime-avibactam was observed in 98% (44/45) and 40% (18/45) of the variants, respectively.

We witnessed a frequent enzymatic trade-off, with many mutations enhancing resistance to one antibiotic at the expense of susceptibility to another, highlighting the importance of finely tuned molecular interactions for effective hydrolysis activity. These trade-offs between mutation-driven resistance and compensatory susceptibility shape the clinical impact of KPC variants (2) and highlight the need for accurate rapid detection tests. Indeed, false-negative or false-positive test results could lead to non-optimal choices of treatment or the delayed implementation of infection control measures.

The two LFIAs evaluated here (Resist-5 O.K.N.V.I and NG-Test Carba 5) had the highest sensitivity scores (88% and 87%, respectively). Hong et al. evaluated Resist-5 O.K.N.V.I on three KPC variants (KPC-2, KPC-3, and KPC-4) and reported that they were correctly detected (13), whereas Ding et al. reported the misdetection of 3 of 6 variants (KPC-33, KPC-71, and KPC-76) by the NG-Test Carba 5 (3, 14). In our study, the six variants (including five with “ceftazidimase” profiles) not detected by NG-Test Carba 5 had mutations in the omega-loop that might disrupt the conformation of the protein, thereby affecting its detection. KPC-37 (with W165R and F207L mutations) had a KPC-like profile and was accurately detected by other diagnostic tests, including the Resist-5 O.K.N.V.I. test. This double mutation may alter the conformation of the protein, potentially disrupting the epitope recognized by NG-Test Carba 5. Similarly, the KPC-32 variant (combining D179Y, T243M, and H274Y mutations) was not detected by LFIA or any of the other diagnostic tests evaluated. Shields et al. reported the clinical emergence of this variant after 10 days of treatment (15, 16). False-negative results for these rapid diagnostic tests could lead to inappropriate antibiotic management.

The results of carbapenem hydrolysis tests were well correlated with resistance profiles, with most positive tests obtained for the KPC-like group. None of the variants with ESBL-like or ceftazidimase profiles were detected by these tests. Four variants with a “KPC-like” profile were not detected by the two carbapenem hydrolysis tests but were correctly detected by the other methods, including growth on selective ChromID Carba Smart medium (Table 2 and Table S3).

Oueslati et al. (17) studied five clinical variants of KPC (KPC-5, KPC-6, KPC-12, KPC-31, and KPC-33) and reported a sensitivity of about 75% for hydrolysis tests, whereas Bianco et al. (18) were unable to detect any of the three variants they tested (KPC-14, KPC-31, and KPC-33) in hydrolysis tests (i.e., Rapidec Carba NP test). Another study evaluated the ability of tests to detect two variants, KPC-14 and KPC-28 (19), both of which have an “ESBL-like” phenotype. As in our study, these strains were not detected by carbapenem hydrolysis tests.

Variants with a “KPC-like” profile and 80% of those with “mixed” profiles grew on ChromID Carba Smart medium, which was designed for use in screening for carbapenem resistance. By contrast, none of the variants with “ESBL-like” or “ceftazidimase” profiles grew on this medium. This suggests that all KPC-like variants retain some degree of resistance to carbapenems, even if this cannot be detected in hydrolysis tests. Conversely, this screening test cannot detect variants susceptible to carbapenems with ESBL-like or ceftazidimase profiles.

Interestingly, the “ESBL-like” variants were recovered on ChromID ESBL agar and with the Beta Lacta hydrolysis test. However, the “ceftazidimase” variants did not grow on ChromID Carba Smart or ChromID ESBL and were not detected by the Beta Lacta test, which may result in their being overlooked by microbiologists. This result may reflect the use of a cephalosporin-like molecule resembling cefotaxime, rather than ceftazidime in the test. Ceftazidimase mutants remain susceptible to cefotaxime, resulting in an absence of hydrolysis and a negative test result. This lack of detection may account for their undetected spread in the hospital environment, potentially leading to outbreaks (20). Most of these variants had a high MIC for ceftazidime-avibactam. The use of a specialized medium, such as Chromatic Super CAZ/AVI, might therefore be useful for the detection of fecal carriage of these variants (21, 22). Eight of the nine variants (89%) with “ceftazidimase” profiles were detected on this medium.

In cases of uncertainty, further molecular tests could be conducted (3, 14, 23). Most rapid molecular diagnostic tests rely on probe binding or melting curve analyses to improve specificity, raising concerns about a possible lack of detection due to mutations or large indels. We therefore assessed the detection of all variants with point mutations, deletions (up to 11 AA), and insertions (up to 15 AA) by real-time PCR-based methods (GenExpert and FilmArray). Both methods successfully detected all variants, indicating that the targeted amplified regions probably lie outside the principal sites of mutation (3, 14).

Our results suggest that LFIA has the best overall sensitivity, regardless of the observed phenotype. The sensitivity of selective agar media and hydrolysis tests is directly linked to the phenotype observed. It is not, therefore, reasonable to recommend a single test. A combination of tests is likely to be more effective for detection. The accurate detection of KPC variants is essential to guide treatment and control measures in healthcare settings, to prevent hospital outbreaks (20). KPC variants are not expected to spread, particularly as reversion to the ancestral profile (carbapenem resistance) has been shown to occur. Wang et al. described such reversion for a KPC-2-producing *K. pneumoniae* isolate that mutated to KPC-33 (D179Y) under ceftazidime-avibactam selection pressure and reverted to the ancestral enzyme profile after the introduction of imipenem (8). It is for this reason that we believe, contrary to other authors, that the detection of KPC variants is important, whatever their resistance profile (24).

This study had several limitations. We chose 45 KPC variants to represent the diversity of mutations found in clinical variants. However, with over 200 clinical variants identified to date, we cannot rule out the possibility that some of the other variants have different phenotypes with different impacts on the ability of tests to detect them. Additional resistance mechanisms may also accumulate in clinical isolates, altering resistance phenotypes and complicating the detection of suspected KPC beta-lactamase variants. Resistance to ceftazidime or ceftazidime-avibactam may alert microbiologists to the possibility of such variants being present, but it may not always be sufficient for detection.

In this context, we propose a strategy to help microbiologists optimize the detection of KPC variants depending on the situation:

– When screening for fecal carriage, the use of multiple selective media is essential. ChromID Carba Smart agar supports the growth of “ancestral KPC,” “KPC-like,” and some “mixed” variants but not that of “ESBL-like” and “ceftazidimase” variants. ChromID ESBL media should be used for the detection of “ESBL-like” variants, and Chromatic Super CAZ/AVI media can identify all ceftazidime-avibactam-resistant isolates (or those with MIC close to the breakpoint), including most “ceftazidimase,” “ESBL-like,” and some “mixed” variants, enhancing sensitivity.
– “Ancestral KPC” are easy to detect by antibiotic susceptibility testing, thanks to their resistance to almost all beta-lactams and also because most of the tests were designed to detect these enzymes. Unexpected resistance to ceftazidime or ceftazidime-avibactam can alert microbiologists to the likelihood of KPC variants being present as well as specific phenotypic profiles, such as “KPC-like,” “ESBL-like,” “ceftazidimase,” or “mixed” profiles. The most appropriate test depends on the phenotype. However, LFIA can be performed as a first-line test, capable of detecting most variants. For “KPC-like” or “mixed” variants, carbapenem hydrolysis tests may also be used, but their sensitivities are lower. In cases of an “ESBL-like” phenotype with suspicion of a KPC variant (due to high-level resistance to ceftazidime ± avibactam, or the presence of epidemiological factors such as travel to or hospitalization in a KPC-endemic region), we recommend using an LFIA test. If a “ceftazidimase” profile is observed, LFIA is the only test that can be used, as other methods typically fail. In cases of a high degree of suspicion despite negative results, PCR should be performed for the detection of mutated *bla*_KPC_.

Our results stress the need for microbiologists to be vigilant when faced with Enterobacterales potentially expressing KPC variants with atypical resistance profiles.

## MATERIALS AND METHODS

### Isolates and mutagenesis

KPC variants (*n* = 45) from two genetic backgrounds, KPC-2 or KPC-3 (differing by the H274Y mutation), including ancestral enzymes (KPC-2 and KPC-3) and three non-clinical variants presenting deletions in the omega-loop of the KPC protein were used (C7 and C1 with deletions of 2 and 11 amino acids, respectively, relative to KPC-2, and C6 with a deletion of four amino acids relative to KPC-3). Seven of these variants were constructed for a previous study (25), and 38 were newly constructed for this study (Table 1).

The variants studied were chosen so as to represent the diversity of clinical variants and specifically to include variants at the most frequently mutated sites of KPC. For example, D179 was mutated in 39 of 242 (16%) of the clinical variants, and insertions or deletions in the omega-loop_164-179_ or Loop_267–275_ were found in 34 of 242 (14%) and 94 of 242 (39%) of the variants, respectively. We also captured a maximum of clinical variant diversity by choosing variants with mutations of less frequently mutated residues outside of the main mutated loops (e.g., M49, A62, G89) (Table 1). Briefly, mutagenesis was performed with overlapping primers containing the mutated codon and amplification of the whole plasmid with Phusion High-Fidelity DNA Polymerase and pBR322-KPC-2 or pBR322-KPC-3 as the template, followed by *Dpn*I digestion, gel purification, and ligation with T4 ligase (New England BioLabs). All variants were in a similar genetic background (pBR322—a low to medium copy number plasmid) and expressed in *Escherichia coli* TOP10 (26).

MICs were determined in triplicate by broth microdilution with Sensititre plates (ESB1F, FRAM2GN, and EUMDRXXF) (Thermo Fisher Scientific) as specified by the manufacturer, with interpretation according to EUCAST guidelines (11, 27). *E. coli* ATCC 25922 was used as a reference.

### PCA to classify KPC variants on the basis of susceptibility profiles

Antibiotic susceptibility testing was performed by the disk diffusion method on Mueller-Hinton agar. The diameter of the inhibition zone for representative beta-lactams (amoxicillin, amoxicillin-clavulanate, cefotaxime, ceftazidime, piperacillin-tazobactam, ertapenem, imipenem, and meropenem) was used for PCA. This method was used to explore the underlying structure and patterns within the data set and to capture the maximum variance in the data. It enhances data visualization, revealing hidden patterns and clusters that it would otherwise be challenging to detect. PCA was performed with R software (available at https://www.r-project.org/) and the FactoMineR package (available from https://cran.r-project.org/web/packages/FactoMineR/index.html).

### Rapid diagnostic tests

We evaluated the detection capacities of two LFIAs targeting specific epitopes of KPC proteins: the Resist-5 O.K.N.V.I. assay (Coris BioConcept, Gembloux, Belgium) and NG-Test Carba 5 (NG Biotech, Guipry, France); two hydrolysis tests detecting carbapenemase activity: the Beta Carba test (Bio-Rad, Marnes-la-Coquette, France) and Rapidec Carba NP (bioMérieux Marcy l'Etoile, France); and one detecting ESBL activity: the Beta Lacta test (Bio-Rad, Marnes-la-Coquette, France). All these tests were evaluated with one to three colonies from a fresh culture on trypticase soy agar in accordance with the manufacturers’ recommendations (Table S1).

### Sensitivity of detection on selective agar media

Three selective media—ChromID Carba Smart, ChromID ESBL (Bio Mérieux, France), and Chromatic Super CAZ/AVI (Liofilchem, Italy) used to screen for carbapenem resistance, ESBL-producing Enterobacterales, and ceftazidime-avibactam resistance—were tested with inocula of 10^2^ and 10^5^ CFU after 24 hours of incubation at 35 ±2°C (Table S1). For that, the strains were suspended in normal saline to a 0.5 McFarland standard (~2 × 10⁸ CFU/mL) and then subjected to serial 10-fold dilutions up to ~2 × 10^4^ CFU/mL. A 10 µL aliquot from the appropriate dilutions was spread onto the three different selective media. The experiments were performed in triplicate.

### Molecular tests

We evaluated the detection capacity of two rapid PCR-based methods: the Xpert Carba-R assay (Cepheid, Sunnyvale, USA) and BioFire FilmArray Blood culture BCID2 (BioFire Diagnostics, USA). A suspension of 10^5^ CFU/mL was prepared to conduct molecular tests.

## SUPPLEMENTAL MATERIAL

Table S1 aac.00082-25-s0001.docx Principles and targets of the phenotypic tests applied to the 45 KPC beta-lactamase variants.

Table S2 aac.00082-25-s0002.xlsx Results of antibiotic susceptibility tests performed by the disk diffusion method with representative beta-lactams and used for PCA analysis.

Table S3 aac.00082-25-s0003.xlsx Results of the phenotypic and genotypic tests on 45 KPC beta-lactamase variants, including the ancestral variants KPC-2 and KPC-3.

ASM does not own the copyrights to Supplemental Material that may be linked to, or accessed through, an article. The authors have granted ASM a non-exclusive, world-wide license to publish the Supplemental Material files. Please contact the corresponding author directly for reuse.
