# Supplementary material for: Evaluation of phenotypic and genotypic methods for detecting KPC variants
Source: Antimicrob Agents Chemother. 2025 Apr 3;69(5):e00082-25. doi: 10.1128/aac.00082-25 (PMC12057356; doi:10.1128/aac.00082-25)
Supplement: Table S1 — Principles and targets of the phenotypic tests applied to the 45 KPC beta-lactamase variants. [file aac.00082-25-s0001.docx]

|  | RESIST-5 O.K.N.V.I. | NG-Test CARBA 5 | BETA CARBA TEST | RAPIDEC CARBA NP | BETA LACTA TEST | CHROMID ESBL | CHROMID CARBA SMART | Chromatic Super CAZ/AVI |
| --- | --- | --- | --- | --- | --- | --- | --- | --- |
| Principle underlying the detection test | Detection of the binding of a monoclonal antibody to the KPC enzyme (amino acids G149 to R182) (1) | Detection of the binding of a monoclonal antibody to the KPC enzyme (amino acids G29 to R270). (2) | Detection of the hydrolysis of a β-lactam (a broad-spectrum cephalosporin stable against ESBLs and cephalosporinases) by the carbapenemase (3) | Detection of the hydrolysis of chromogenic imipenem by the carbapenemase (4) | Detection of the hydrolysis of HMRZ-86 (a chromogenic cephalosporin) by the ESBL enzyme (5) | Selective culture of strains harboring an ESBL stable against cefpodoxime (6) | Selective culture of strains harboring a carbapenemase | Culture of strains resistant to the ceftazidime-avibactam combination |
|  |  |  |  |  |  |  |  |  |

Supplemental Table 1: Principles and targets of the phenotypic tests applied to the 45 KPC beta-lactamase variants

References

1. Bogaerts P, GLUPCZYNSKI G, HUANG TD, Mertens P, OTE I, Leclipteux T. Method and device for detecting a carbapenemase-producing enterobacteriaceae [Internet]. EP3280815B1, 2020 [cité 29 déc 2024]. Disponible sur: https://patents.google.com/patent/EP3280815B1/en

2. Boutal H, Naas T, Devilliers K, Oueslati S, Dortet L, Bernabeu S, et al. Development and Validation of a Lateral Flow Immunoassay for Rapid Detection of NDM-Producing Enterobacteriaceae. J Clin Microbiol. 23 juin 2017;55(7):2018‑29.

3. Bernabeu S, Dortet L, Naas T. Evaluation of the β-CARBA^TM^ test, a colorimetric test for the rapid detection of carbapenemase activity in Gram-negative bacilli. J Antimicrob Chemother. 1 juin 2017;72(6):1646‑58.

4. Poirel L, Nordmann P. Rapidec Carba NP Test for Rapid Detection of Carbapenemase Producers. J Clin Microbiol. sept 2015;53(9):3003‑8.

5. Renvoisé A, Decré D, Amarsy-Guerle R, Huang TD, Jost C, Podglajen I, et al. Evaluation of the βLacta Test, a Rapid Test Detecting Resistance to Third-Generation Cephalosporins in Clinical Strains of Enterobacteriaceae. J Clin Microbiol. 21 déc 2020;51(12):4012‑7.

6. Réglier-Poupet H, Naas T, Carrer A, Cady A, Adam JM, Fortineau N, et al. Performance of chromID ESBL, a chromogenic medium for detection of Enterobacteriaceae producing extended-spectrum β-lactamases. J Med Microbiol. 2008;57(3):310‑5.
